# Validity and reliability of the Portuguese version of the rapid estimate of adult literacy in dentistry: REALD-29 PT

**DOI:** 10.1186/s12903-022-02289-w

**Published:** 2022-06-28

**Authors:** Helder Costa, Odete Amaral, João Duarte, Maria José Correia, Nélio Jorge Veiga, Joaquin Francisco López-Marcos

**Affiliations:** 1 Faculty of Medicine, University of Salamanca, Salamanca, Spain; 2 Universidade Católica Portuguesa, Faculty of Dental Medicine, Centre for Interdisciplinary Research in Health (CIIS), Viseu, Portugal; 3 Health School of Viseu, Polytechnic Institute of Viseu, Viseu, Portugal; 4 Surgery Department, Faculty of Medicine, University of Salamanca, Salamanca, Spain

**Keywords:** REALD-30, REALD-29PT, Elderly, Oral health literacy

## Abstract

**Background:**

Health literacy is a main factor in health for its improvement, allowing the individuals to have a greater capacity to engage and participate in collective health promotion actions. The evaluation of functional health literacy is essential to determine the ability that each individual has to understand basic health information. The present study aimed to perform the translation and cross-cultural adaptation of the Rapid Estimate of Adult Literacy in Dentistry-30 to the Portuguese language and test the reliability and validity of this version.

**Methods:**

After translation and cultural adaptation, the instrument was applied to a group of individuals that participate in the program Atividade Senior, developed by the municipality of Viseu, Portugal. The final sample was composed by 206 participants that accepted responding to the translated version of the instrument. Statistical validation was accomplished to complete the process and obtain the final instrument. One question was removed for the creation of the final instrument with 29 questions, therefore being named Rapid Estimate of Adult Literacy in Dentistry-29 PT.

**Results:**

The Rapid Estimate of Adult Literacy in Dentistry-29 PT presented good internal reliability. Cronbach’s alpha ranged from 0.89 to 0.90 when words were deleted individually. The analysis of test–retest reliability revealed excellent reproducibility. We can verify that the Rapid Estimate of Adult Literacy in Dentistry-29 PT scale for assessment of oral health literacy among older adults presents an acceptable internal consistency, with a global Cronbach´s alpha of 0.894.

**Conclusions:**

The new scale can be applied to assess oral health literacy among older Portuguese adults, presenting an acceptable internal consistency and is validated to assess oral health literacy and is crucial in epidemiological studies.

## Background

Health literacy is essential to empower the community to improve their health and quality of life levels, allowing individuals to have a greater capacity to be involved and participate in collective health promotion actions [1]. Low levels of health literacy can be the main barrier to the adoption of adequate daily habits, reflecting the increased demand for health services and increasing spending on medical care [2]. It is fundamental to evaluate functional health literacy to determine the capacity that each individual has to understand basic health information [3, 4].

In recent years there has been an increase in studies developed about health literacy [5], however the development of studies dedicated to oral health literacy is very recent. Oral health literacy can be described as the level that an individual has to obtain, process and understand the basic oral and craniofacial information and health services necessary to make appropriate oral health decisions [6].

Low oral health literacy leads to less frequent adoption of positive and adequate oral health habits and therefore worse oral health outcomes [7]. On the other hand, an increase in the level of oral health literacy is associated with better communication between the patient and the oral health professional, which promotes a reduction in anxiety levels during the dental appointment and less reluctance to receive medical help [8].

Studies are not consensual regarding the association between oral health literacy levels and the individual's oral health condition. Nevertheless, it is important to assess functional health literacy to determine capacity. There are several instruments in the literature that can be applied to measure the level of oral health literacy. The most used requires word recognition, as is the case of the Rapid Estimate of Adult Literacy in Dentistry (REALD-30) [9].

The REALD-30 is a specific tool to assess the level of oral health literacy through the recognition of words ordered in a list with varying degrees of difficulty [4]. This instrument is easy and quick to apply in clinical practice, which is why most studies carried out use it [9, 10]. Despite the fact that REALD-30 is a word recognition tool and evaluates only some of the skills in terms of individual literacy, studies show that it is highly correlated with functional health literacy as well as having good psychometric properties [4, 11].

Several studies indicate that there is a relationship between the level of oral health literacy and oral health status, the adoption of positive oral health behaviors and satisfaction with oral health care services [12, 13]. For this reason, it is imperative to have valid and credible instruments capable of measuring the level of oral health literacy.

Therefore, the aim of this study consisted in the translation and cross-cultural adaptation of REALD-30 into the Portuguese language (REALD-29 PT) and test the reliability and validity of this version.

## Methods

The REALD-30 is a specific instrument for assessing the level of literacy among adults regarding oral health through the recognition of words referring to etiology, anatomy, prevention, and treatment of specific oral conditions. The instrument is composed by 30 words that should be read aloud by the participant to the interviewer. The list of words is arranged in ascending order of difficulty based on both the average word length, number of syllables and the level of difficulty of combining sounds. For each word pronounced correctly, one point is assigned to the REALD-30 score and zero is recorded when the pronunciation is incorrect. The total score is obtained by summing the scores and ranges from 0 (lowest degree of literacy) to 30 (highest degree of literacy) [14].

In this study the translation, adaptation, and validation of the REALD-30 to the Portuguese population was accomplished in two complementary phases: (i) translation and cultural adaptation of the questionnaire and (ii) statistical validation. The translation and cultural adaptation were performed to obtain a questionnaire equivalent to that developed in the original country regarding contents and semantics. For this adaptation, the translation-retroversion method for bilingual individuals was applied. The translation process began with two independent translations from the original REALD-30 scale by two translators, both Portuguese and fluent in English. The translated version was reviewed by an English native Dentist in Portugal. Retroversion was accomplished by an independent translator, who didn’t have any knowledge or contact with the original version in English. The original and retranslated versions were compared to assess the content of items and finally the correction of technical terms was performed.

The REALD-30 in the Portuguese language was administered to 206 individuals that participated in the Atividade Senior program organized by the municipality of Viseu, Portugal, that agreed in responding to the REALD-30 Portuguese version. To each participant, cards with the written words were presented and read out loud to the interviewer. Furthermore, the time each participant took to complete reading the word list was recorded. Data collection was accomplished between January 2019 and December 2019.

Statistical analysis of the database was performed using the IBM-SPSS® 24.0 and Factor 10.8. An exploratory factorial analysis was developed, based on the following analysis details. Most of the items were categorical or dichotomous items, and the test–retest reliability of questionnaire was assessed by calculation of the Cronbach´s alpha coefficient. A coefficient higher than 0.6 indicates an acceptable consistency and the coefficient for each item is presented as a median with a 95.0% confidence interval.

All procedures performed were in accordance with the ethical standards of the institutional research committee and with the 1964 Helsinki declaration and its later amendments or comparable ethical standards. The research was approved by the Health Ethics Committee of the Universidade Católica Portuguesa (Approval number 100). Patients participating in the study signed a written Informed Consent Form confirming their willingness to participate in this study.

## Results

The scale was validated in a sample consisting of 206 participants with an average age of 72.3 ± 5.4 years, most of them female (n = 149, 72.3%). Since all the participants answered all questions of the REALD-30 scale 100% valid cases were considered.

REALD-30 scores obtained had a mean score of 19.25 ± 5.794. The average execution / response time of 30 words was 1 min and 34 s, with a minimum of 48 s and maximum of 3 min and18 s.

The word “sugar” on the scale was excluded at the time of validation since it presented 0 variance, meaning it was a homogeneous response. Thus, when validating the REALD-30 scale for Portugal, we considered only 29 items, and therefore propose that the Portuguese version is described as REALD-29 PT.

The statistics (mean, confidence interval, asymmetry, and shortness) of each item of the REALD-29 PT scale are presented in Table 1. According to the values of asymmetry (higher than 3) and kurtosis (higher than 7) we could eliminate items if necessary.

**Table 1:** Statistics of each item of the REALD-29 PT scale

Variable	Item (Portuguese language)	Item (English language)	Mean	Confidence Interval	Variance	Skewness	Kurtosis
a2	Fumar	Smoking	0.971	(0.94–1.00)	0.028	−5.628	29.521
a3	Fio dentário	Floss	0.811	(0.74–0.88)	0.153	−1.594	0.533
a4	Escovar	Brush	0.903	(0.85–0.96)	0.088	−2.735	5.449
a5	Polpa	Pulp	0.854	(0.79–0.92)	0.124	−2.019	2.062
a6	Fluor	Fluoride	0.587	(0.50–0.68)	0.242	−0.357	−1.868
a7	Aparelho	Braces	0.947	(0.91–0.99)	0.051	−3.992	13.866
a8	Genética	Genetics	0.796	(0.72–0.87)	0.162	−1.477	0.176
a9	Restauração	Restoration	0.922	(0.87–0.97)	0.072	−3.171	8.013
a10	Bruxismo	Bruxism	0.689	(0.61–0.77)	0.214	−0.822	−1.322
a11	Abcesso	Abscess	0.563	(0.47–0.65)	0.246	−0.256	−1.930
a12	Extração	Extraction	0.830	(0.76–0.90)	0.141	−1.767	1.110
a13	Dentadura	Denture	0.801	(0.73–0.87)	0.159	−1.515	0.289
a14	Esmalte	Enamel	0.927	(0.88–0.97)	0.068	−3.304	8.869
a15	Dentição	Dentition	0.660	(0.58–0.74)	0.224	−0.680	−1.535
a16	Placa	Plaque	0.942	(0.90–0.98)	0.055	−3.790	12.303
a17	Gengiva	Gengiva	0.820	(0.75–0.89)	0.147	−1.677	0.805
a18	Mal oclusão	Maloclusion	0.238	(0.16–0.31)	0.181	1.237	−0.472
a19	Incipiente	Incipient	0.510	(0.42–0.60)	0.250	−0.039	−1.994
a20	Caries	Caries	0.723	(0.64–0.80)	0.200	−1.003	−0.994
a21	Periodontal	Periodontal	0.121	(0.06–0.18)	0.107	2.330	3.409
a22	Selante	Sealant	0.709	(0.63–0.79)	0.206	−0.923	−1.147
a23	Hipoplasia	Hypoplasia	0.311	(0.23–0.39)	0.214	0.822	−1.322
a24	Halitose	Halitosis	0.549	(0.46–0.64)	0.248	−0.196	−1.957
a25	Analgesia	Analgesia	0.199	(0.13–0.27)	0.159	1.515	0.289
a26	Celulite	Cellulitis	0.704	(0.62–0.79)	0.208	−0.898	−1.193
a27	Fistula	Fistula	0.563	(0.47–0.65)	0.246	−0.256	−1.930
a28	Temporo mandibular	Temporo mandibular	0.044	(0.01–0.08)	0.042	4.487	18.037
a29	Hiperemia	Hyperemia	0.374	(0.29–0.46)	0.234	0.524	−1.722
a30	Apicectomia	Apicoectomy	0.141	(0.08–0.20)	0.121	2.076	2.293

After performing the Bartlett`s sphericity test (Bartlett's statistic) a value of 1871.0 (df = 406; *p* < 0.001) was obtained. The Kaiser–Meyer–Olkin (KMO) test was calculated as 0.87453 which represents a good classification and the BC Bootstrap 95% confidence interval of KMO was 0.877–0.878. All these values indicate that the level of acceptance for the validation process was very positive. With these values an exploratory factor analysis was performed, and a single factor structure resulted with eigenvalues greater than one (eigenvalue = 2985). The parallel analysis reinforces the existence of a single factor with an explained variance percentage of 12.8%.

A Cronbach’s alpha coefficient ranging between 0.5 and 0.7 is generally considered satisfactory for comparisons between groups, while values higher than 0.85 are sufficiently reliable for comparisons on the individual level.

Cronbach´s alpha for 29 items was 0.894, varying between 0.887 and 0.894 when words were excluded individually, that is, the instrument had good internal reliability with 29 items. Therefore, the REALD-29 PT shows satisfactory psychometric properties for use among Portuguese adults, as presented in Table 2.

**Table 2:** Values of Cronbach´s alpha coefficient por the REALD-29 PT (n = 206)

Variable	Item (Portuguese Version)	Item (English language)	R	R without item	α without item
a2	Fumar	Smoking	0.3179	.294	.894
a3	Fio dentário	Floss	0.4841	.430	.891
a4	Escovar	Brush	0.2867	.240	.894
a5	Polpa	Pulp	0.5942	.553	.889
a6	Fluor	Fluoride	0.5893	.529	.889
a7	Aparelho	Braces	0.3850	.353	.893
a8	Genética	Genetics	0.6454	.602	.888
a9	Restauração	Restoration	0.4971	.462	.891
a10	Bruxismo	Bruxism	0.5737	.516	.890
a11	Abcesso	Abscess	0.6649	.613	.887
a12	Extração	Extraction	0.5782	.533	.889
a13	Dentadura	Denture	0.5352	.484	.890
a14	Esmalte	Enamel	0.3969	.358	.893
a15	Dentição	Dentition	0.6182	.564	.888
a16	Placa	Plaque	0.3279	.293	.894
a17	Gengiva	Gengiva	0.5564	.508	.890
a18	Mal oclusão	Maloclusion	0.4648	.404	.892
a19	Incipiente	Incipient	0.5502	.485	.890
a20	Caries	Caries	0.5977	.544	.889
a21	Periodontal	Periodontal	0.2615	.208	.895
a22	Selante	Sealant	0.5566	.499	.890
a23	Hipoplasia	Hypoplasia	0.5423	.482	.890
a24	Halitose	Halitosis	0.5678	.505	.890
a25	Analgesia	Analgesia	0.4157	.356	.893
a26	Celulite	Cellulitis	0.6262	.574	.888
a27	Fistula	Fistula	0.5854	.525	.889
a28	Temporomandibular	Temporo mandibular	0.2536	.220	.894
a29	Hiperemia	Hyperemia	0.5044	.438	.891
a30	Apicectomia	Apicoectomy	0.3880	.335	.893

## Discussion

In Portugal, the lack of oral health literacy is a serious public health issue. We still find in our population concerning levels of lack of health literacy with consequences for systemic health in general, and oral health in particular [14]. Therefore, the application of proper scales to assess oral health literacy is important for the establishment of proper health education strategies in the community. Although the literature doesn´t present consistent results in terms of impact of oral health literacy in the oral health status it is widely accepted that lower literacy is linked to problems with the use of preventive services, delayed diagnoses of medical conditions, poor adherence to medical instructions, poor self-management skills, increased mortality risks, poor health outcomes, and higher health care costs [7]. The lack of consistent results demonstrating these links may be related to poor quality of the study design and instruments available to assess oral health literacy [15].

This is, as far as we could ascertain, the first validation of this scale for Portugal and presents as an important tool to define oral health literacy criteria and to understand what must be developed in terms of oral health behavior education, promotion, and motivation at a community level.

REALD-29 PT demonstrated a considerably high internal consistency, as the Cronbach's alpha coefficient was 0.89, similar to that published by other authors for the Portuguese Brazilian version or the original English version [4, 16].

The REALD-29 PT demonstrated acceptable psychometric properties and proved to be a quick, simple, and reliable instrument to measure oral health literacy among older Portuguese adults. It turns out to be an efficient instrument for screening on an individual level to identify individuals with a low degree of oral health literacy, allowing oral health professionals to adjust their communication strategies for each patient specifically.

The instrument can be applied in association with other indicators to better assess the oral health literacy among the population, providing information to health administrators and policymakers, supporting the development of appropriate educational and oral health promotional approaches and prevention strategies.

One of the limitations found during the research, more specifically, during data collection, was the difficulty in having participants understand what was supposed to be done which limited the selection of the participants. This fact might be due to an overall low literacy level of the studied population. However, the present research presents an acceptable sample number of responses for the validation of the scale since it is very close to the original as well as the Brazilian Portuguese instrument validations [4].

Having a Portuguese version of the scale, REALD-29 PT is an essential tool to assess oral health literacy among the Portuguese population and understand the type of adaptations towards oral health programs that in the future.

## Conclusion

The REALD-29 PT scale to assess oral health literacy among older Portuguese adults presents an acceptable internal consistency and proved to be a reliable and valid tool, self-reported to identify the level of oral health literacy. REALD-29 PT is a validated scale to assess oral health literacy and is crucial for epidemiological studies and the improvement of the oral heath interventions in specific communities.

## Data Availability

The data used to generate and support the findings of this study are available from the corresponding author upon request.

## Abbreviations

REALD: Rapid estimate of adult literacy in dentistry
PT: Portugal
